# Biogenic zinc selenide nanoparticles fabricated using *Rosmarinus officinalis* leaf extract with potential biological activity

**DOI:** 10.1186/s12906-023-04329-6

**Published:** 2024-01-04

**Authors:** Shahram Ahmadi Somaghian, Seyedeh Zahra Mirzaei, Mohammad Ebrahim Khosravi Shakib, Abdolrazagh Marzban, Sarah Alsallameh, Hamed Esmaeil Lashgarian

**Affiliations:** 1 Razi Herbal Medicines Research Center, Lorestan University of Medical Sciences, Khorramabad, Iran; 2 Medical Biology Research Center, Kermanshah University of Medical Sciences, Kermanshah, Iran; 3 Department of Medical Laboratories Techniques, College of Health and Medical Techniques, Gilgamesh Ahliya University Gau, Baghdad, 10022 Iraq; 4 Department of Medical Biotechnology, Lorestan University of Medical Sciences, Khorramabad, Iran

**Keywords:** Zinc selenide nanoparticles, Biological activity, Green synthesis, *Rosmarinus officinalis* L

## Abstract

Zinc selenide nanoparticles (ZnSe) are semiconductor metals of zinc and selenium. ZnSe NPs are advantageous for biomedical and bio-imaging applications due to their low toxicity. ZnSe NPs can be used as a therapeutic agent by synthesizing those using biologically safe methods. As a novel facet of these NPs, plant-based ZnSe NPs were fabricated from an aqueous extract of *Rosmarinus officinalis L*. (RO extract). Physiochemical analyses such as UV-visible and FTIR spectroscopy, SEM-EDX and TEM Imaging, XRD and DLS-Zeta potential analyses confirmed the biological fabrication of RO-ZnSe NPs. Additionally, Ro-ZnSe NPs were investigated for their bioactivity. There was an apparent peak in the UV-visible spectrum at 398 nm to confirm the presence of ZnSe NPs. FTIR analysis confirmed RO-extract participation in ZnSe NPs synthesis by identifying putative functional groups associated with biomolecules. TEM and SEM analyses revealed that RO-ZnSe NPs have spherical shapes in the range of 90–100 nm. According to XRD and EDX analysis, RO-ZnSe NPs had a crystallite size of 42.13 nm and contain Se and Zn (1:2 ratio). These NPs demonstrated approximately 90.6% antioxidant and antibacterial activity against a range of bacterial strains at 100 µg/ml. Antibiofilm activity was greatest against *Candida glabrata* and *Pseudomonas aeruginosa* at 100 g/ml. Accordingly, the IC_50_ values for anticancer activity against HTB-9, SW742, and HF cell lines were 14.16, 8.03, and 35.35 g/ml, respectively. In light of the multiple applications for ZnSe NPs, our research indicates they may be an excellent option for biological and therapeutic purposes in treating cancers and infections. Therefore, additional research is required to determine their efficacy.

## Introduction

Nanotechnology has gained significant attention in various fields, including medicine and materials science, food, agriculture, etc. NPs possess unique properties such as large surface areas to volumes, quantum confinement, and surface plasmon resonance [1–3]. Therefore, they are promising candidates for drug-delivering and catalyzing applications.

Conventional methods used to synthesize NPs often involve chemical processes that can be expensive, energy-intensive, and environmentally hazardous. As a result, researchers have turned their focus toward better alternatives that utilize natural sources like plant metabolites, bacterial and fungal bio-products and other bio-based catalysts [3].

Multiple biological systems, including plants, algae, bacteria and fungi have been used to produce eco-friendly metal NPs. The process of synthesizing metal based NPs from plant metabolites is significantly more controllable and easier to scale up than theses NPs synthesized from bacteria and fungi. This approach of NPs fabrication has diverse biomedical and environmental applications, including anti-inflammatory properties, therapeutics, drug delivery systems, medical diagnosis, imaging, and sensors [4, 5].

ZnSe is a compound semiconductor material that has unique optical properties and potential applications in optoelectronic devices and biomedical imaging technologies [6]. However, synthesizing ZnSe NPs using conventional chemical processes can be often for other applications except biomedical and pharmaceutical fields. Therefore, researchers have turned their focus toward greener alternatives that utilize natural sources like plant extracts to fabricate ZnSe NPs with more compatibility to biological conditions [7]. Green-synthesized ZnSe NPs have unique optical and electronic properties, making them an appealing alternative for various applications, including biomedical imaging technologies, drug delivery systems, and therapeutics. Further investigations are required to fully understand the functions and potential applications of green-synthesized ZnSe NPs [4, 7].

*Rosmarinus Officinalis L*. plant is a member of the mint family *Lamiaceae*, perennial, evergreen and fragrant plant with needle-shaped leaves that is mainly found in the Mediterranean region [3]. *Rosmarinus officinalis* (commonly known as rosemary) is an herb widely recognized for its medicinal properties attributed to its phytochemical composition [1]. The leaf extract contains various bioactive compounds such as polyphenols and flavonoids with antioxidant potential. These bioactive compounds have been found to have therapeutic potential in traditional medicine with a variety of applications in infectious diseases and metabolic disorders.

Despite the increasing interest in the green synthesis of ZnSe NPs, most studies focus on their physicochemical properties. Additionally, *Rosmarinus officinalis* leaf extract has been demonstrated to generate green NPs based on metals. Therefore, this study sought to examine the plant synthesis of ZnSe NPs by rosemary for the first time. Further, this study is noteworthy because of the evaluation of the resulting ZnSe NPs for antimicrobial, cytotoxic, and antioxidant activities.

## Methods and materials

### Chemicals and cell lines

Chemicals of high purity grade, such as zinc nitrate hexahydrate and sodium selenite, were supplied by Merck Chemical Company. Ascorbic acid, 3-(4’5-dimethylthiazol)-2’5-diphenyl tetrazolium bromide (MTT), crystal violet, and 2’2-diphenyl-1-picrylhydrazyl (DPPH) were of high quality as received. Three cell lines, human skin fibroblasts (HFFF2-C163), human bladder cancer HTB-9 (ATCC 5637), and human colon cells SW742 (CVCL_3884), were prepared from the cell collection of the Pasteur Institute, Tehran, Iran. The bacterial and fungal strains were purchased from the microorganism collection of the Iranian Research Organization for Science and Technology (IROST).

### Plant preparation and extraction method

*Rosmarinus officinalis* L. leaves were prepared from medicinal plant herbarium at Razi Herbal Medicines Research Center, Lorestan, Khorramabad, Iran. The herbarium voucher for this specimen of rosemary was obtained from the electronic herbarium of Jihad University of Karaj, Iran and given as HerID: 312. Rosemary aqueous extraction was carried out according to a method described by Daghestani et al. (2020) [8]. The biomass was washed with distilled water (DW) and dried at ambient temperature. The dry matter was then poured into 100 ml (DW) and heated in water bath-sonicator at 65 ° C for 45 min. The sample was then separated by the Whatman filter paper (no.1) from the biomass and was used to synthesize nanoparticles.

### Qualitative photochemical assessments

#### Total flavonoid assay

The total flavonoid content of the samples was determined using a colorimetric method with aluminum chloride. For this, 100 µg of dried extract was dissolved in 1 ml acetone and added in 1 ml of aluminum chloride solution (3%). Then 0.25 mL potassium acetate (120 mM) was added to the reaction solution and allowed to stand for 15 min at room temperature. A standard curve was constructed based on serial dilutions of rutin as a flavonoid indicator. The absorbance of the solution was measured at 415 nm based on the λ_max_ absorbance of rutin. The results were expressed in terms of rutin content. The experiment was performed in three replicates and the data were expressed as mean ± SD [9].

#### Total phenolic assay

Total phenolic content as the equivalent of gallic acid (GA) per gram of dried plant extract was estimated based on the Folin-Ciocalteu (FC) phenolic assay. First, a standard curve was provided using gallic acid methanol solution (80%) as a known phenolic compound. One milliliter of FC reagent (diluted as 0.1 in DW) was added to 2 ml of plant extract. The sample was mixed with 1 ml sodium carbonate (Na_2_CO_3,_ 1 M) and was incubated for 120 min. The absorbance of the reaction sample was determined at 760 nm [9]. Total phenolic compounds were detected by adding 1 ml of ferric chloride solution (1%) to 1 ml of RO extract (50 µg/ml). After 10 min, the appearance of a green-yellow coloration indicates the presence of phenolic compounds [10].

#### Total terpenoids assay

The terpenoids in the RO extract were measured using a colorimetric method based on vanillin sulfuric acid (V-SA). For this, 0.5 ml of RO extract (50 mg/ml) was added to 2 ml of V-SA (1:1) containing 1 mg/ml vanillin and incubated at 55 °C for 30 min. The absorbance of the sample solution was determined at 550 nm using a spectrophotometer. Quercetin was used as a control for the preparation of a standard curve [11].

Additionally, a qualitative total terpenoids assay was performed by blending 2 ml chloroform with 5 ml RO extract (500 µg/ml) and 3 ml concentrated sulfuric acid. The presence of terpenoids was indicated by the formation of a reddish brown interface of the solution [10].

### Biosynthesis of ZnSe NPs by RO extract

To fabricate ZnSe NPs, firstly, 0.5 g of RO extract was dissolved in 20 ml of DW. In a separate container, 0.4 g of Zinc acetate dehydrate and 0.25 g of sodium selenite (Na_2_SeO_3_) were dissolved in 80 ml of DW. The reaction mixture was placed on a magnetic stirrer and RO extract was slowly added while stirring continuously. After 30 min, small amounts of ethanol were added dropwise to the sample to promote the precipitation of ZnSe NPs in the mixture. The sample was maintained under the same conditions to complete NPs formation for 12 h. Finally, the produced ZnSe NPs were collected by centrifugation at 12,000 rpm for 15 min. The precipitate was purified by washing using DW and dried at 60 °C in an oven.

### RO-ZnSe physicochemical characteristics

Several spectroscopic and microscopic methods were used to analyze the elemental composition, precise shape, and other physicochemical features of the prepared ZnSe NPs. Light absorbance and optical properties of ZnSe NPs were studied using UV-VIS spectroscopy (Jenway, model 6505, UK) with a resolution of 1 nm in the 350–600 nm range. Fourier Transform Infrared (FTIR) spectrometry was exploited to evaluate the functional groups of bioactive metabolites to prepare ZnSe NPs on an FTIR system (Bruker Tensor 27, Optics GmbH). Morphological and chemical studies were performed using FE-SEM imaging and energy dispersive X-ray (EDX) analysis in a scanning emission microscopy (SEM) system (TESCAN MIRA3, Czech Republic). Crystallographic analyses were performed on an X-ray diffractometer apparatus (Bruker, D8 Advance, Germany) and X-ray powder diffraction (XRD). Using XRD data, particle size distribution, crystallite size, and phase type were determined using ImageJ software Ver. 2 (NIH, USA) and XPert HighScore plus Ver. 2.2 software, respectively.

### Anti-proliferative potential of RO-ZnSe NPs

The cell viability was determined using the quantitative MTT colorimetric technique. HTB-9, SW741, and HFF-2 cell lines were grown at a density of 5 × 10^3^ cells/well in a 96-well plate and treated for 48 h at exposure to different doses of RO-ZnSe NPs. The cell culture media was then removed from the wells, 100 µl of fresh medium containing 10 µg/ml MTT were added to the wells and the test plates were incubated for 4 h at 37 °C. After discarding the supernatant, 100 µl of DMSO was applied to each well to dissolve the formazan crystals. The number of living cells was proportional to the formazan produced in DMSO, which was quantified at 490 nm using a microplate reader (ELISA MAT2000, DRG Instruments, Marburg, Germany). Cell viability was determined as percentages as follows:

1 $${\rm{Viability}}\,\left( \% \right)\,{\rm{ = }}\,{{{\rm{Absorbance}}\,{\rm{of}}\,{\rm{treated}}\,{\rm{sample}}} \over {{\rm{Absorbance}}\,{\rm{of}}\,{\rm{control}}}}\, \times \,{\rm{100}}$$

### Antimicrobial activity assessment

Antimicrobial analysis was performed against various human pathogens: Two Gram-negative bacteria, *Pseudomonas aeruginosa* (*P. aeruginosa*) (ATCC27853) and *E. coli* (ATCC 25,922); two Gram-positive bacteria, *Staphylococcus aureus* (*S. aureus*) (ATCC 12,600), *Staphylococcus saprophyticus* (*S. saprophyticus*) (ATCC35552); two fungi *Candida albicans* (*C. Candida albicans*) (ATCC 1031) and *Candida glabrata* (*C. glabrata*) (ATCC 48,435) were selected to evaluate the antimicrobial activity of RO-ZnSe-NPs. For this purpose, the antimicrobial activity of RO-ZnSe-NPs against bacterial and fungal pathogens was carried out by the agar well diffusion method. Chloramphenicol (30 µg/ml) and amphotericin B (30 µg/ml) were positive controls for bacteria and fungi, respectively. Bacterial and fungal cells were spread on the agar plates using a sterile swab; a 6 mm punch was used to create the wells in the agar. Then 40 µl of each dilution of RO-ZnSe-NPs (12.5, 25, 50 and 100 µg/ml) were added to each well. After 24 h of incubation at 37 °C, the growth inhibition diameters were determined with a ruler. In addition, the MIC of RO-ZnSe NPs was examined by the microdilution assay in 96-well plates, and the triphenyl tetrazolium chloride (TTC) reagent was used to evaluate the viability of the microorganisms. All the experiments were carried out in triplicate assay.

### Biofilm inhibition assay

This experiment was performed to evaluate biofilm inhibition of RO-ZnSe NPs against the aforementioned pathogens in 96-well plates. The bacterial cells were adjusted to 1.5 × 10^8^ cfu/ml (0.5 McFarland cell densities) in each well and 200 µl of LB-broth medium, containing 100, 50 and 25 and 12.5 µg/ml of RO-ZnSe NPs for all pathogens. The treated samples were incubated at 37 °C for 24 h in a bacterial incubator. After that, the plates were extensively rinsed with distilled water to remove non-adherent bacterial cells. Subsequently, adhered biofilms were stained with crystal violet (0.1% v/v) for 5 min, and then excess dyes were washed with DW. To examine the stained biofilms, 200 µl of acetic acid were poured into each well and agitated slowly for 5 min. The absorbance was measured at 570 nm using an ELISA plate reader. Antibiofilm potential was presented as percentage by the following formula (2):

2 $${\rm{Antibiofilm}}\,{\rm{activity}}\,\left( \% \right)\,{\rm{ = }}\,{{{\rm{Control}}\left( {{\rm{OD}}} \right)\, - \,{\rm{treated}}\left( {{\rm{OD}}} \right)} \over {{\rm{Control}}\left( {{\rm{OD}}} \right)}}\, \times \,{\rm{100}}$$

### DPPH scavenging assay

The antioxidant capacity of RO-ZnSe NPs was examined using the DPPH inhibition assay compared to ascorbic acid (AA). Different concentrations of ZnSe nanoparticles were combined with a DPPH reagent (0.15 mM in methanol). The reaction was allowed to proceed in darkness for 30 min. AA was taken as a positive control. The DPPH scavenging efficiency was calculated using the following Eq. (3):

3 $${\rm{Scavenging}}\,{\rm{efficacy}}\,\left( {\rm{\% }} \right)\,{\rm{ = }}\,{{{\rm{Blank}}\left( {{\rm{A0}}} \right)\, - \,{\rm{Sample}}\left( {\rm{A}} \right)} \over {{\rm{Blank}}\left( {{\rm{A0}}} \right)}}\,{\rm{ \times }}\,{\rm{100}}$$

### Statistical analysis

The statistical analyses were conducted with GraphPad Prism 7 (version 7). To assess significant differences among the samples, a fully randomized design was used through Tukey’s multiple range tests and analysis of variance (ANOVA). The data is given in the form of mean values together with their corresponding standard deviations. The statistical significance was determined based on P-values < 0.05.

## Results and discussion

### Phytochemical characterization of RO-extract

**Fig. 1:**
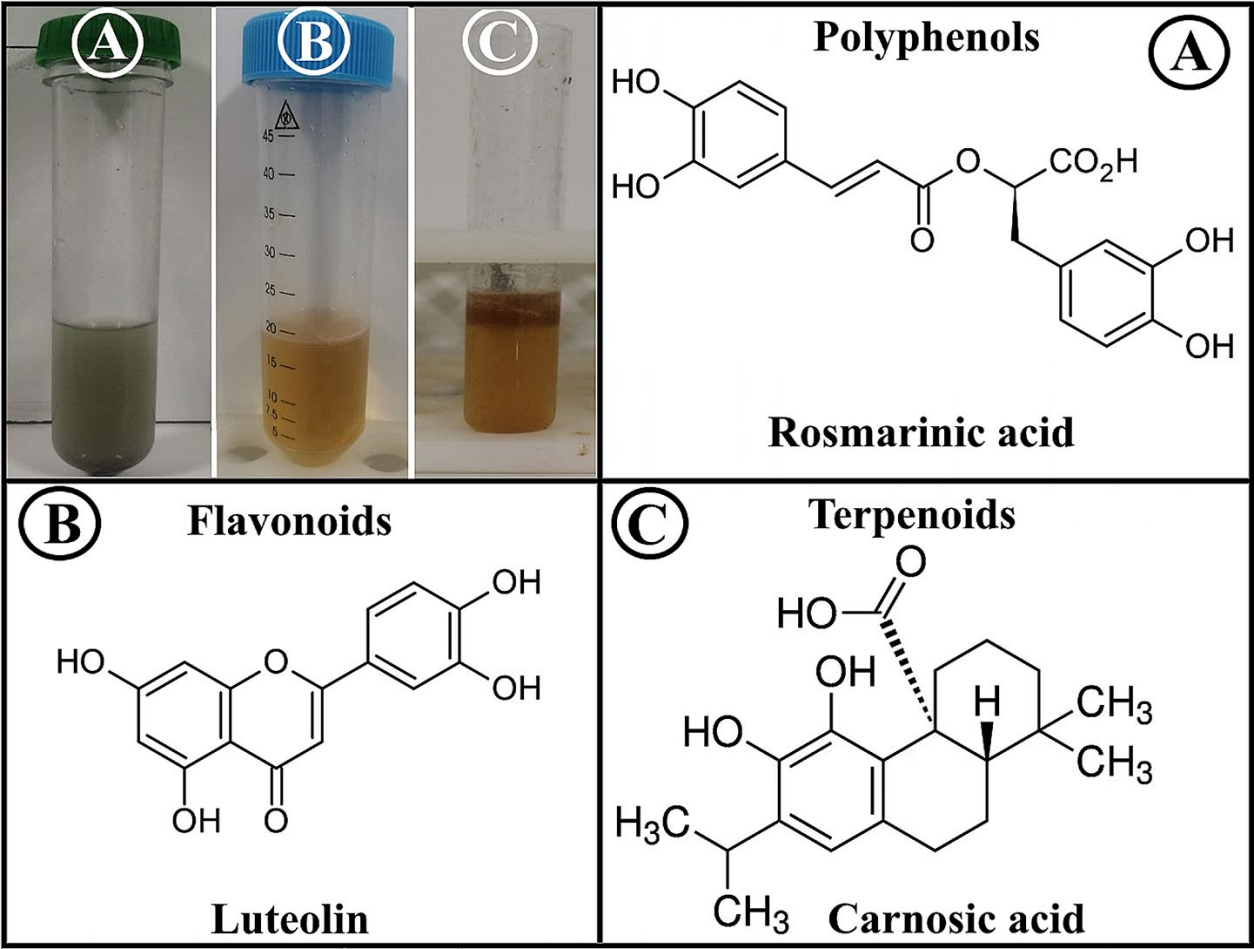
Qualitative detection of phytochemical contents in RA extract. (**A**) Total polyphenols, grey to blue (Rosmarinic acid), (**B**) Total Flavonoids (Leteolin) and (**C**) Total terpenoids (Carnosic acid)

The presence of bioactive phytochemicals in the RO extract was confirmed using colorimetric methods. Figure 1 confirmed the presence of Polyphenols, flavonoids and terpenoids in the RO extract. Quantitative studies showed that the RO extract used in this study contained flavonoids (18.2 µg/ml), polyphenols (36.5 µg/ml), and terpenoids (69.06 µg/ml). Studies have shown that *R. officinalis* contains a variety of bioactive metabolites, including flavonoids such as luteolin, diosmin, apigenin, and glucoside derivatives [12]. The second group included terpenoids, including carnosic acid and its alcoholic derivatives. Polyphenols, especially rosmarinic acid derivatives, were the second most important type [13]. The main phytochemicals responsible for producing most metal NPs are polyphenols, flavonoids, terpenoids and their subfamilies [14]. According to the findings, some biomolecules found in plant extracts may act as reducing agents in synthesizing metal NPs. Due to its biocompatibility, low cytotoxicity and environmental friendliness, the green production of metal NPs using plant extracts is a potential technology for use in medicine [15]. In addition, plant bio-variations offer a variety of biochemical properties and represent a unique source for NPs synthesis [16]. As a result, RO-ZnSe NPs derived from RO extract show higher bioactivity, which can be attributed to various active metabolites that reduce, cap and stabilize the NPs.

### Characterization of biosynthesized ZnSe NPs

#### Optical properties of RO- ZnSe NPs

**Fig. 2:**
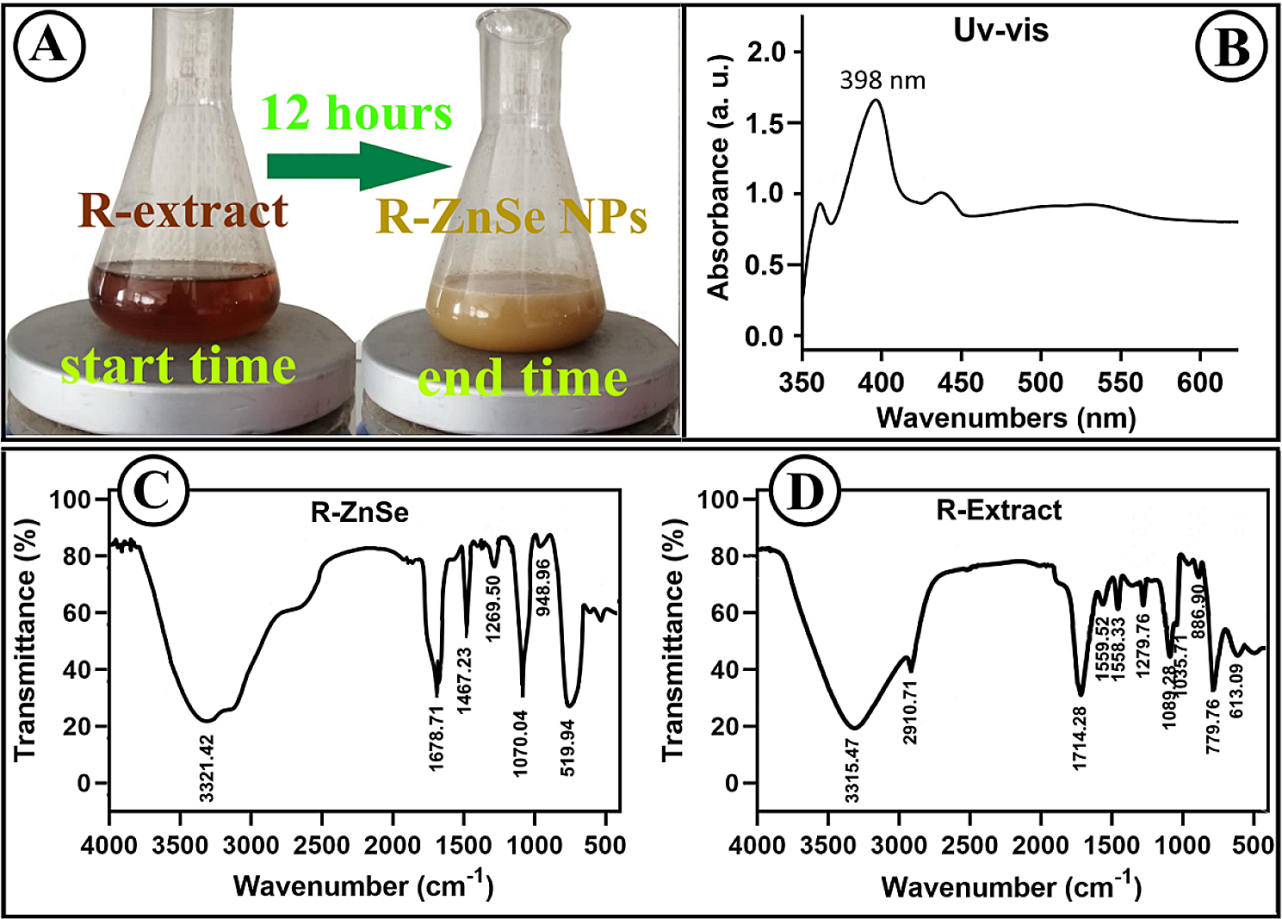
ZnSe NPs formation procedure. (**A**) Color change of the reaction mixture during NPs synthesis. (**B**) UV–Visible spectra of biosynthesized ZnSe NPs by RO extract (**C**) FTIR spectra of (**C**) RO-synthesized ZnSe NPs and (**D**) Aqueous extract of RO at the range of 500–4000 cm^− 1^

The formation of ZnSe NPs was confirmed by the color shift of the solution, with the RO extract changing from red to brown (Fig. 2). According to the UV-Vis spectrum, a distinct peak at 398 nm corresponds to the formation of ZnSe NPs by the RO extract. Although the search results do not directly compare the absorption ranges of green and chemically prepared ZnSe NPs, they provide some insight into their differences. Mirzaei et al. (2021) reported biologically produced ZnSe NPs with absorption edges of 375 nm and band gaps of 3.3 eV [17]. Hong et al. (2020) showed that ZnSe NPs absorb UV-Vis light at wavelengths shorter than 460 nm, the wavelength at which bulk ZnSe absorbs UV-Vis light [18]. In their study, the authors proposed the biosynthesis of ZnSe NPs by increasing the Zn/Se ratio, thereby shifting the wavelength of the UV-Vis absorption edge to 420 nm. The blue shifts can be attributed to the broadening of the absorption curves of ZnSe NPs toward shorter wavelengths. ZnSe NPs can exhibit a wide range of absorptions and peak intensities depending on the process and surrounding medium used in their preparation. For example, depending on their size and shape, aqueous RO extract can successfully synthesize ZnSe NPs in 300–400 nm [19].

#### FTIR spectroscopy analysis

To identify the functional groups in RO extract that contribute to the capping and stability of ZnSe NPs, FTIR spectroscopic analysis was performed. As shown in Fig. 2C, the stretching peak at 3315.4 cm^− 1^ in the spectrum of ZnSe NP has been attributed to O-H in the RO bioactive metabolites, with a slight shift to 3321.4 cm^− 1^ due to steric hindrance [20]. A bordering peak at 2910.7 cm^− 1^ is attributed to stretching symmetric hydroxyl in the carboxylic groups of RO metabolites [1]. A stretching peak at 1714.2 cm^− 1^ is attributed to the amine groups of RO metabolites. Vibrations of the amine (N-H) groups were responsible for a stretching peak at 1678.7 cm^− 1^ [4]. In addition, the shift and weakening of a strong peak at 1089.2 cm^− 1^ can be attributed to the deformation of C-O-C stretching in the structure of RO metabolites compared to 1070.0 cm^− 1^ in the spectrum of ZnSe NPs. The biosynthesis of ZnSe NPs by RO metabolites was inferred from a stretching peak at 479.1 cm^− 1^ [6, 21].

#### FE-SEM and TEM imaging of RO-ZnSe NPs

**Fig. 3:**
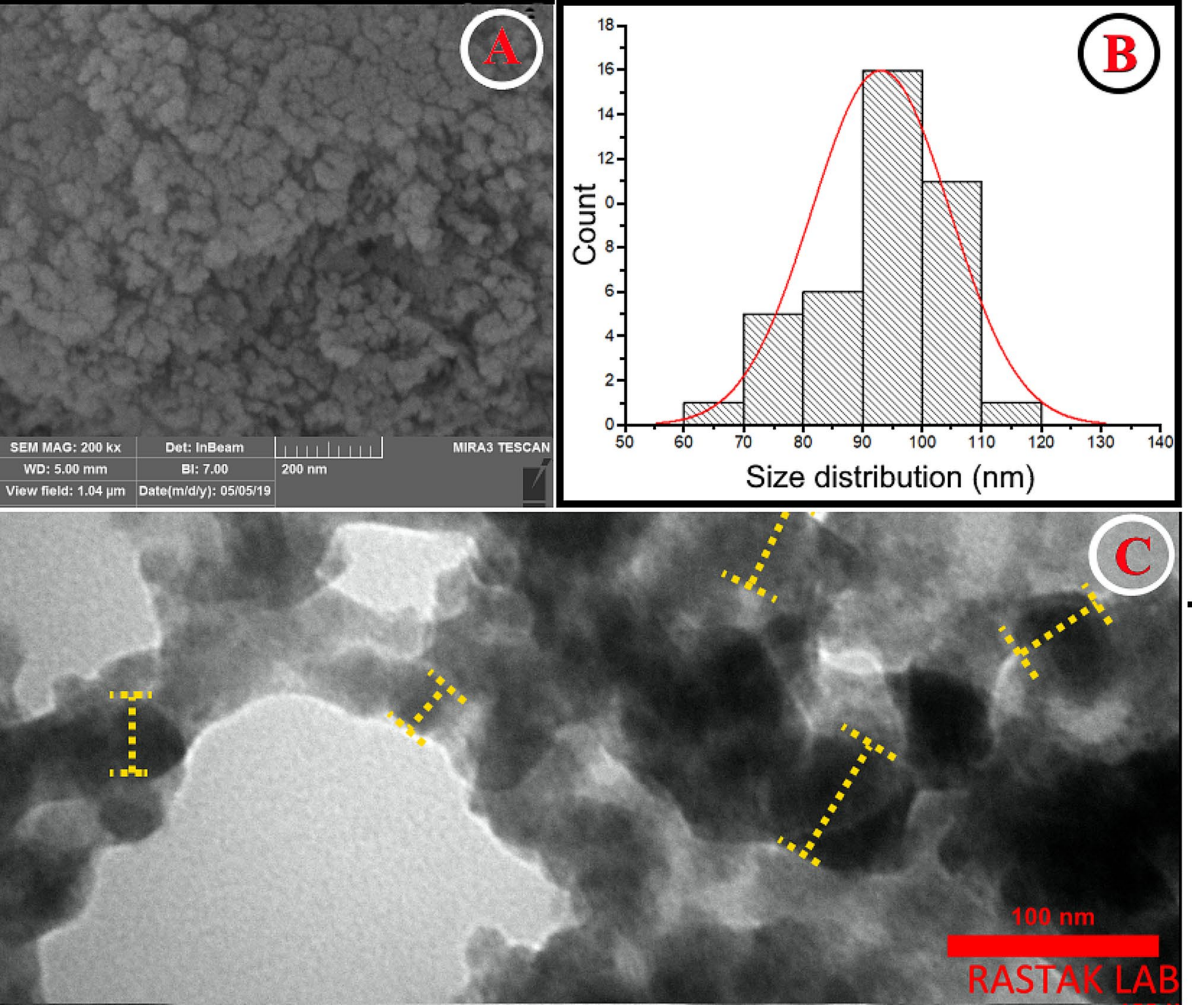
Microscopic morphology RO-ZnSe NPs. (**A**) SEM and (**B**) Particle size distribution based on SEM imaging scale and (**C**) TEM image of ZnSe NPs synthesized

The ZnSe NPs’ morphological features were examined through FE-SEM analysis. Figure 3A reveals the various shapes and sizes of ZnSe NPs with some agglomerations in their structure. Based on the size distribution pattern calculated on spherical and agglomerated nano-sized particles, the average size of ZnSe NPs was 90–100 nm (see Fig. 3B). Additionally, the TEM analysis confirmed that RO metabolites incorporated the biogenic ZnSe NPs. Based on Size labeling on the TEM image, the particle size is less than 100 nm, which could approve the size predictions calculated from the SEM analysis (Fig. 3C).

#### XRD and energy-dispersive X-ray spectroscopy of RO-ZnSe NPs

**Fig. 4:**
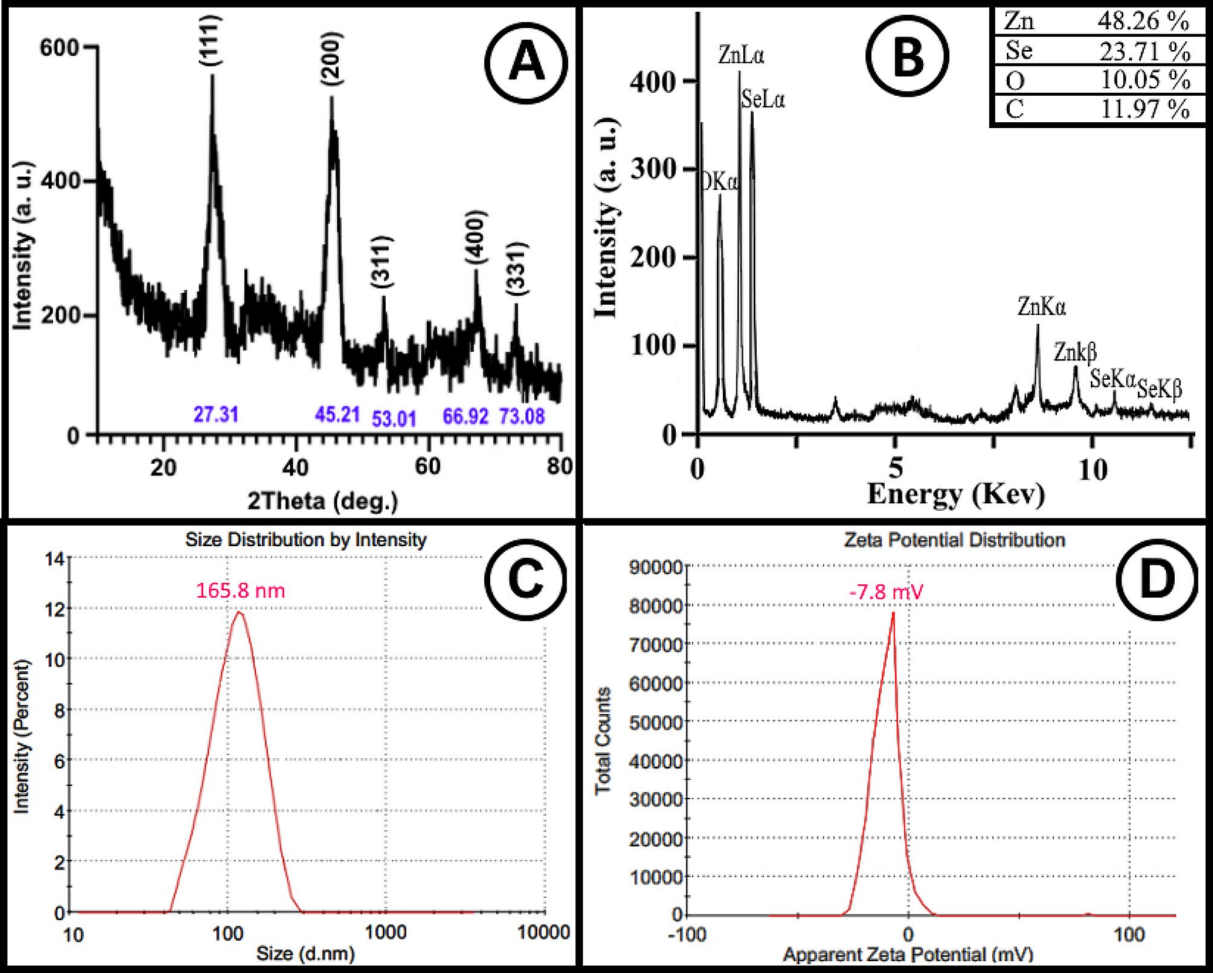
Crystallography and elemental analysis (**A**) XRD patterns, (**B**) EDX compositional analysis of RO-ZnSe NPs (**C**) Particle size distributions (DLS) and (**D**) Zeta potential within the surface of RO-ZnSe NPs in aqueous solution

The crystallite phases and elemental abundances of the RO-ZnSe NPs were determined by XRD and EDX, respectively. The XRD pattern of ZnSe NPs synthesized from RO extract is depicted in Fig. 4A. The crystallographic database indicates that distinct peaks at 2 angles of 27.31°, 45.21°, 53.01°, 66.92°, and 73.08° correspond to the normal crystal planes (111), (200), (311), (400), and (331), respectively. The structure of these patterns has been predicted as a face-centered cubic (FCC) [17, 22]. As shown in Fig. 4B, the XRD spectrum revealed sharp peaks indicating the presence of zinc and selenium in the RO-ZnSe nanocrystals. This is consistent with the XRD analysis reported by Hernández et al. (2014) [23]. According to the Scherrer equation, the crystallite size of RO-ZnSe NPs was calculated to be 42.13 nm, agreeing with the size range from TEM and SEM data.

Energy dispersive X-ray (EDX) microanalysis is recognized as a spectroscopy instrument for detecting metal contaminants in a variety of biological applications, including diagnosis and research. EDX is a surface microanalysis technique for determining the elemental composition of nanoparticles [24]. The EDX profile of RO-ZnSe NPs is depicted in Fig. 4B. Here, the main elements of RO-ZnSe NPs nanoparticles are represented by intense peaks of Zn, Se, O, and C. As anticipated, the EDX pattern confirms the high purity of the resulting ZnSe NPs fabricated by RO extract.

### Particle size and surface charge distribution assay

The size distribution of dispersed particles in an aqueous phase is considered a critical factor in estimating stability, drug release, accumulation, tissue diffusion and other physicochemical behaviors mimicking physiological conditions of the living body [7, 16, 20]. In this case, DLS determined an average particle size distribution of 165 nm for the hydrodynamic diameters of particles suspended in the aqueous phase (Fig. 4c). According to the literature, NPs 100–200 nm in size were effective for tissue-specific distribution, while those larger than 200 nm showed low tissue diffusion [25, 26]. The zeta potential is a quantitative indicator of the electrostatic forces between NPs based on their surface charge, with implications for attraction or repulsion. It is critical in upholding colloid stability. Typically, zeta potentials of NPs ranging from − 10mV to + 10mV qualify as nearly neutral [26]. Besides, NPs with zeta potentials exceeding + 30 mV and falling below − 30 mV are classified as strong cations and anions, respectively [27, 28]. On the other hand, the zeta potential of NPs can affect their ability to cross membranes, and it is commonly observed that cationic particles pose a greater risk in terms of damaging cell walls [26]. Therefore, a significant correlation exists between the zeta potential and the biological effectiveness of NPs. As seen in Fig. 4d, zeta potential value of -7.8 mV suggests that the surface charge of RO-ZnSe NPs is nearly neutral. Consequently, the antibacterial and antioxidant properties indicate excellent outcomes considering the NPs size.

### Antiproliferative outcomes of RO-ZnSe NPs

**Fig. 5:**
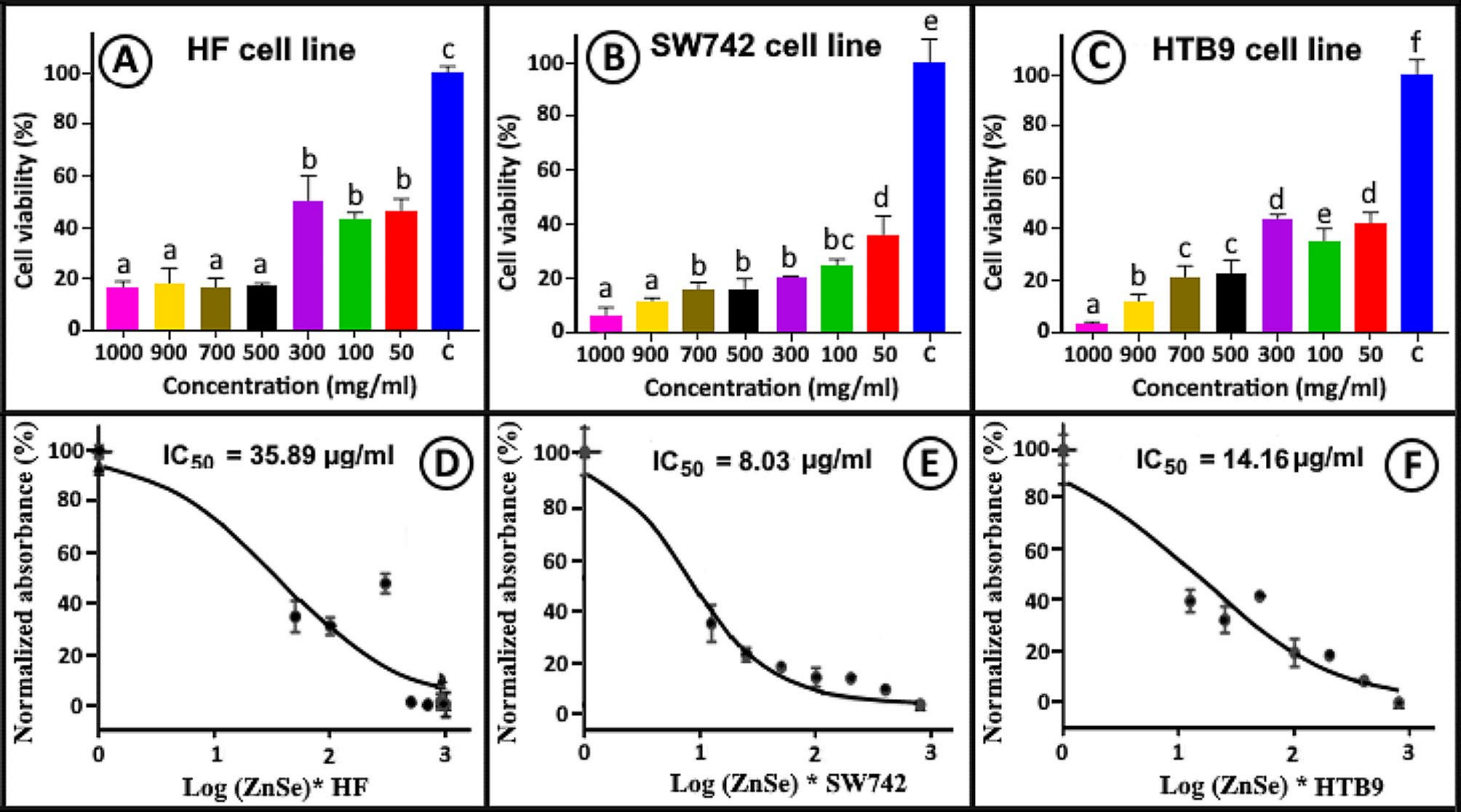
Viability of the cell lines. (**A** and **D**) viability and IC50 value of HF (normal) cell line, (**B** and **E**) viability and IC50 value of SW742 (colon) cell line and (**C** and **F**) viability and IC_50_ value of HTB9 (bladder) cell line at 50-1000 µg/ml of ZnSe NPs. Different superscript indicates significant differences between groups

The antiproliferative activity of RO-ZnSe NPs was examined on two cancer cell lines: HTB9 human bladder carcinoma cell line and SW742 human allogeneic colon carcinoma cell lines in comparison with normal fibroblast cells, HF using the MTT assay. Figure 5 depicts the pattern of cell viability at various RO-ZnSe NPs concentrations (50-1000 mg/ml) for tumor and normal cell lines. RO-ZnSe NPs exhibited dose-dependent cytotoxicity against all cell lines, as predicted. However, the IC_50_ values for HF, HTB9, and SW742 were 35.89, 8.03, and 14.16 mg/ml, respectively. Based on the results, it was possible to conclude that the cytotoxic effect of RO-ZnSe NPs has a selective mode for malignant and normal cells, such that a distinction was observed between these cell types.

Moreover, it is intriguing to note that the antiproliferative efficacy of RO-ZnSe NPs is more efficacious against colon cancer (SW742) compared to two other cell lines. Since ZnSe nanostructures, particularly their quantum dots are widely reported as valuable semiconductors for various applications, the therapeutic activities of ZnSe NPs on cancer cells have been the subject of a few studies. Nevertheless, some studies suggest that ZnO NPs, which have a similar structure to ZnSe NPs, produce reactive oxygen species (ROS) that induce apoptosis and cause cytotoxicity [29–31]. In addition, ZnS and other Zn-based NPs have been reported to induce cytotoxicity by generating reactive oxygen species [32]. Consequently, the cytotoxicity of ZnSe NPs on cancer cells may also be related to ROS production. Mirzaei et al. (2021) conducted a study demonstrating that ZnSe NPs synthesized from the seaweed *Gracilaria corticata* demonstrated promising antitumor activity against HTB-9 with an IC_50_ of 11.86 mg/ml [17].

### In vitro antibacterial and antifungal potential of ZnSe NPs

**Fig. 6:**
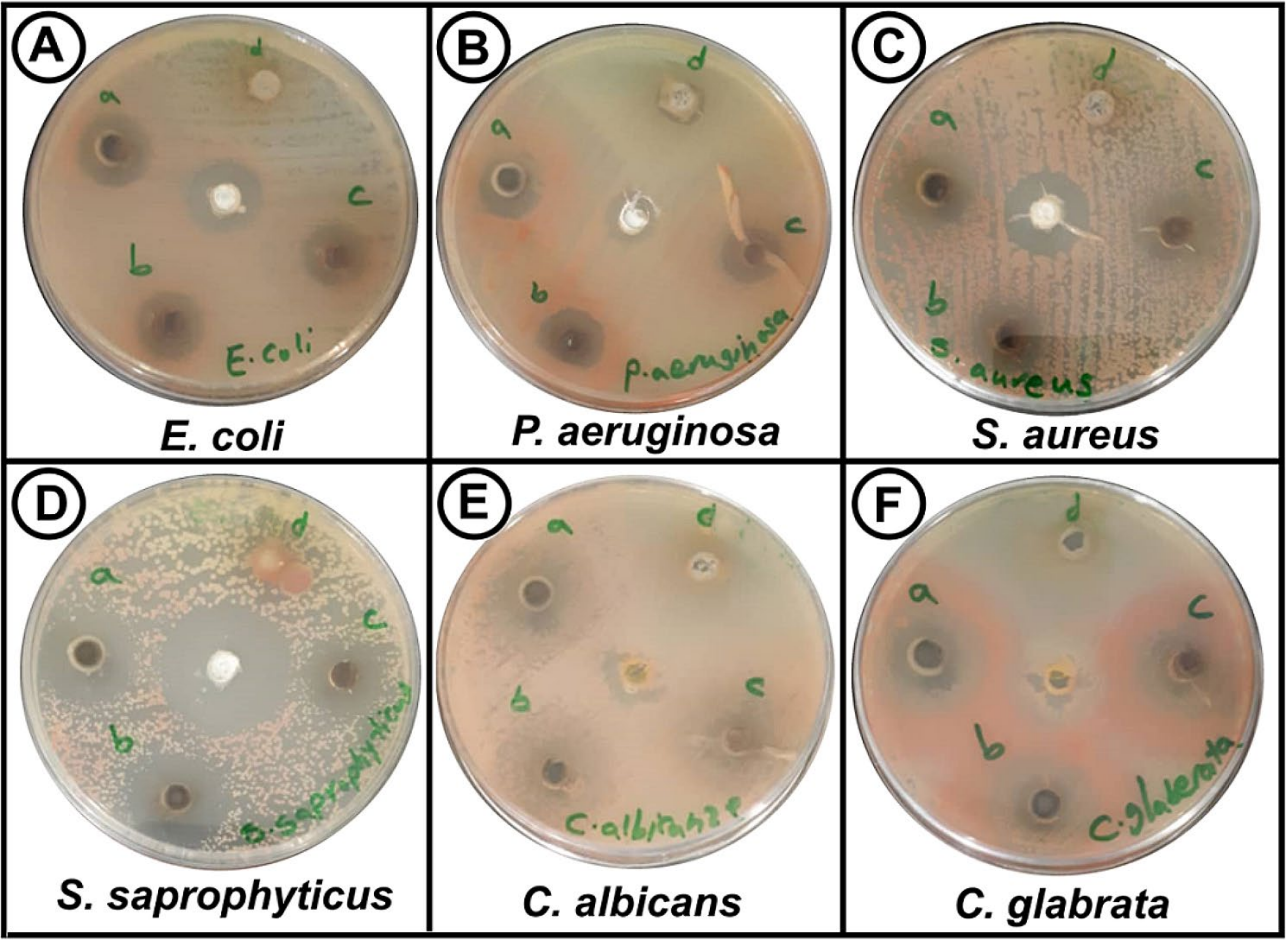
Antimicrobial activity assay based on well-diffusion test. (**A**-**F**) various bacteria and fungi inhibition at four concentrations a: 100, b: 50, c: 25 and d: 12.5 µg/ml. Central wells represent standard antibiotics, chloramphenicol and amphotericin B, for bacteria and fungi, respectively

The antibacterial activity of RO-ZnSe NPs was examined against various pathogens using well-diffusion and MIC methods. As seen in Fig. 6, the growth inhibition zone of bacterial and fungal pathogens at 4 dilutions represents compared with standard antibiotics. In all strains, antimicrobial activities were found to be dose-dependent, so the inhibition zone increased with increasing the doses of RO-ZnSe NPs. Additionally, Table 1 showed precise amounts of zone inhibition diameters and pathogens’ MIC values. The results demonstrated potent activities against gram-positive and fungal strains compared with gram-negative bacteria. In this regard, Khezripour et al. (2019) reported that ZnSe QDs have a remarkable inhibition against various pathogens such as gram-positive and negative bacteria. They stated that the size of NPs could be critical in penetrating efficiency into bacterial membranes [33]. Gupta et al. (2023) showed that green synthesized ZnSe nanostructures had promising photoluminescence potential and antimicrobial activity when annealed at high temperatures after co-precipitation [5]. Souri et al. (2021) produced ZnSe nanocrystals about 2 nm in size, having antibacterial activity against gram-negative bacteria such as *E. coli* and *P. aeruginosa* [34]. Accordingly, Mir et al. (2018) showed a wide-spectrum activity of ZnSe NPs on gram-negative and positive pathogens. They proposed an antimicrobial mechanism by binding ZnSe NPs to biomolecules via amine and thiol groups, disrupting cell function and ultimately causing microbial death [35]. In our previous study, phyco-synthesized ZnSe NPs were examined against various pathogens that had similar outcomes to the present study [17].

**Table 1:** Antimicrobial activity of RO-ZnSe NPs based on agar well-diffusion and MIC values

Microorganism	Growth Inhibition Zone (mm)				MIC
	100	50	25	12.5	µg/ml
*E. coli*	8.5 ± 1.1	7.9 ± 1.0	6.5 ± 0.2	2.1 ± 0.1	100
*P. aeruginosa*	8.9 ± 1.4	7.4 ± 1.2	5.6 ± 0.3	4.5 ± 0.6	100
*S. aureus*	10.1 ± 3.2	8.9 ± 1.6	7.6 ± 1.4	4.4 ± 0.8	75
*S. saprophyticus*	13.3 ± 2.0	10.7 ± 1.1	8.4 ± 1.3	5.0 ± 0.7	50
*C. albicans*	13.6 ± 2.1	9.8 ± 0.9	8.2 ± 0.7	4.1 ± 1.5	50
*C. glabrata*	11.2 ± 1.6	7.3 ± 0.5	5.7 ± 1.1	3.1 ± 0.2	50

### Antibiofilm activity of ZnSe NPs

The efficacy of RO-ZnSe NPs in suppressing biofilm development was evaluated using a colorimetric technique on a 96-well plate after staining with violet crystals. The impact of several concentrations (12.5, 25.50, and 100 µg/ml) of RO-ZnSe NPs on the development of pathogenic biofilms was assessed. The findings in Fig. 6 demonstrate that all strains exhibited the greatest inhibition of biofilm formation when exposed to 100 µg/ml of RO-ZnSe NPs (Table 2). The biofilm-forming capacity of *C. glabrata* and *P. aeruginosa* was significantly reduced by around 90%, whereas *S. saprophyticus* exhibited the lowest level of biofilm inhibition at 63.3%. The present study indicated that the antibiofilm efficacy of RO-ZnSe NPs exhibited a dose-dependent response, with higher concentrations of NPs resulting in less bacterial adherence to surfaces. A major factor contributing to bacterial resistance is the limited ability of antibiotics to penetrate the biofilm matrix. Antimicrobial NPs provide a viable strategy for the eradication of biofilms. Some studies have investigated the antibiofilm activity of zinc-based NPs that have obtained promising results [36]. According to recent studies, it has been shown that NPs of ZnO and Se possess remarkable capabilities in terms of inhibiting the formation of biofilms [37, 38]. However, there is a lack of comprehensive research on the effectiveness of ZnSe NPs in inhibiting the development of biofilms. The mechanism by which ZnSe NPs exhibit anti-biofilm activity is believed to include their interaction with functional groups, including thiol, carboxyl, hydroxyl, and amines. It is believed that these interactions play a critical role in the prevention of the adhesion of bacterial biofilms to surfaces [14, 39, 40].

**Table 2:** Antibiofilm activity (%) at different doses of RO-ZnSe NPs against all tested pathogens

Microorganism	RO-ZnSe NPs (µg/ml)				Antibiotic (µg/ml)	
	100	50	25	12.5		30
*P. aeuroginosa*	87.6 ± 4.7	64.0 ± 3.6	60.1 ± 6.1	30.1 ± 3.2		55.3 ± 5.3
*E. coli*	77.9 ± 6.4	69.9 ± 6.4	57.2 ± 5.3	37.8 ± 4.1		83.4 ± 6.1
*S. saprophyticus*	63.3 ± 7.4	62.1 ± 7.3	48.5 ± 8.0	11.6 ± 2.1		83.8 ± 5.7
*S. aureus*	81.7 ± 6.2	63.4 ± 8.4	60.6 ± 4.6	6.7 ± 1.1		80.7 ± 6.8
*C. albicans*	75.5 ± 6.5	62.1 ± 6.2	49.5 ± 6.3	31.6 ± 3.5		89.3 ± 8.2
*C. glabrata*	90.3 ± 8.4	73.3 ± 5.2	69.2 ± 4.1	50.1 ± 4.0		94.2 ± 8.9

### Antioxidant potential assessment of RO-ZnSe NPs

**Fig. 7:**
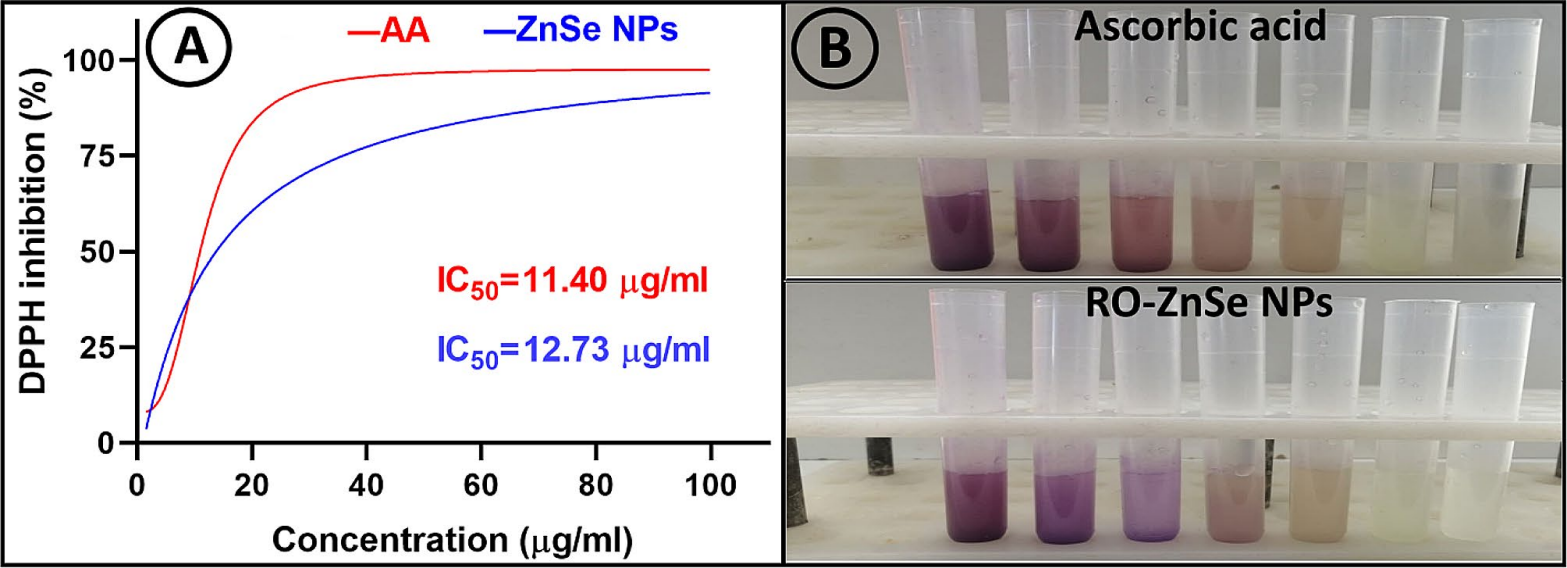
Antioxidant activity of ZnSe NPs. (**A**) DPPH inhibition by ZnSe NPs and AA at concentration range of 0-100 µg/ml. (**B**) Macrodilution experiment of DPPH inhibition by ZnSe NPs and AA

The antioxidant activity of RO-ZnSe NPs was investigated by DPPH inhibition assay. The antioxidant activity was reported as the percentage of ascorbic acid (AA) inhibitory activity. As shown in Fig. 7, the antioxidant activity of RO-ZnSe NPs was dose-dependent, and the DPPH inhibition improved with increasing concentrations of RO-ZnSe NPs. The results showed that ZnSe NPs had a maximum antioxidant activity of 90.6% at 100 g/mL, while AA had a maximum of about 97.5 at 50 µg/mL. Although various studies have documented the antioxidant activity of zinc and selenium NPs, studies on the antioxidant activity of ZnSe NPs are limited [41]. As the literature mentions, NPs with high antioxidant capacity have been reported to significantly alleviate inflammation and improve wound healing [42]. In our previous study, biogenic ZnSe NPs inhibited DPPH by 77.9% at a treatment dose of 30 µg/ml [17]. The antioxidant potential of nanomaterials is influenced by various factors, including modified configuration, physicochemical properties, crystallinity, surface charge, particle size, and surface coating [43]. Therefore, biologically based coatings could have significant and positive antioxidant properties, resulting in enhanced therapeutic effects associated with antioxidant activity [44].

## Conclusion

The preparation of ZnSe nanoparticles was carried out by a biological approach using an aqueous extract of *Rosmarinus officinalis* L. The study results showed that the RO-ZnSe nanoparticles had promising properties in their antibacterial, antifungal, antibiofilm, antioxidant, and anticancer activities. The biological properties of ZnSe nanoparticles have received little attention in previous research efforts. The results of this research indicate that zinc selenide nanoparticles can eradicate a wide range of various pathogenic microorganisms. In addition, the particles possess antioxidant properties that have the potential to alleviate inflammation and protect against the harmful effects of free radical-induced oxidative stress. The biological properties of ZnSe nanoparticles have been overlooked in previous investigations, underscoring the importance of the findings presented in this work. This research also provides one of the few sources of essential information in this particular discipline.

## Data Availability

All data that resulted from this study are fully reported and discussed in the manuscript. Raw data can be received from the corresponding author following the reasonable request.
